# Metformin Improves the Senescence of Renal Tubular Epithelial Cells in a High-Glucose State Through E2F1

**DOI:** 10.3389/fphar.2022.926211

**Published:** 2022-06-23

**Authors:** Dan Liang, Zhiyang Li, Zhaowei Feng, Zhiping Yuan, Yunli Dai, Xin Wu, Fan Zhang, Yuanyuan Wang, Yuxia Zhou, Lingling Liu, Mingjun Shi, Ying Xiao, Bing Guo

**Affiliations:** ^1^ Guizhou Provincial Key Laboratory of Pathogenesis & Drug Research on Common Chronic Diseases, Guizhou Medical University, Guiyang, China; ^2^ Department of Pathophysiology, Guizhou Medical University, Guiyang, China; ^3^ University Town Hospital, Guizhou Medical University, Guiyang, China

**Keywords:** metformin, diabetic kidney disease, DNA damage, renal tubular epithelial cells, fibrosis, E2F1

## Abstract

Diabetic kidney disease is a major cause of chronic kidney condition and the most common complication of diabetes. The cellular senescence participates in the process of diabetic kidney disease, but the specific mechanism is not yet clear. Cell cycle-related protein E2F transcription factor 1 (E2F1) is a member of the E2F transcription factor family, it plays a key role in cellular damage under HG conditions. In this study, we explored whether metformin improves a high-glucose-induced senescence and fibrosis of renal tubular epithelial cells through cell cycle-related protein E2F1. In the *in vivo* experiments, the recombinant adeno-associated virus (AAV-shE2F1) knockdown *E2F1* gene was injected into the tail vein of 16-weeks-old *db/db* mice for 8 weeks. The 16-week-old *db/db* mice were administered metformin (260 mg/kg/d) continuously for 8 weeks. The normal control group (NC) and diabetic model group (DM) were set up simultaneously. Mice renal tubular epithelial cells (mRTECs) were cultured *in vitro*. The cells were randomly divided into the following groups: normal glucose (NG, containing 5.5 mmol/L glucose), high glucose group (HG, containing 30 mmol/L glucose), NG/HG metformin intervention group (NG/HG + Met), NG/HG negative control siRNA transfection group (NG/HG + Control), NG/HG E2F1 siRNA transfection group (NG/HG + siRNA E2F1), HG metformin intervention and overexpression E2F1 plasmid transfection group (HG + Met + overexpress-E2F1). The expression of related indexes were detected by Western blot, real-time polymerase chain reaction (PCR), immunohistochemistry, and immunofluorescence. The results showed that E2F1 knockdown or metformin reduces the degree of renal fibrosis, DNA damage, and cellular senescence in the DM group; metformin also reduced the expression of E2F1. If E2F1 was overexpressed, the effects of metformin in delaying fibrosis and reducing DNA damage and cellular senescence could be weakened. Thus, metformin alleviates high-glucose-induced senescence and fibrosis of renal tubular epithelial cells by downregulating the expression of E2F1.

## Introduction

Aging leads to physiological senescence in an organism, followed by various diseases that further facilitate cellular senescence. The senescent cells accumulate and damage various tissues and organs (Kirkland and Tchkonia, 2017), thus causing loss of function of the body. The production of senescent cells can be triggered by a variety of diseases, such as atherosclerosis, diabetes (Childs et al., 2015), osteoarthritis, and glaucoma (Calcinotto et al., 2019). If these senescent cells are not eliminated promptly, it would further affect the progression of the disease. Obesity and age are the major factors endangering type 2 diabetes, and both increase the burden of cells and promote cellular senescence (Palmer et al., 2019). Especially, obesity increases the level of senescence-associated markers, such as senescence-associated β-galactosidase (SA-β-Gal) activity and the production of senescence-associated secretory phenotype (SASP), such as proinflammatory cytokines interleukin-6 (IL-6) and tumor necrosis factor-α (TNF-α); the level of these inflammatory cytokines accelerate the senescence of neighboring cells (Burton and Faragher, 2018). The study demonstrated that hyperglycemia in type 1 and 2 diabetes models increases the DNA damage response and compromises the DNA repair that results in aging, increased inflammatory phenotype, and renal fibrosis (Kumar et al., 2020). Diabetic kidney disease (DKD) is a severe complication of diabetes mellitus (DM). The typical features of DKD are the thickening of the glomerular basement membrane, the extracellular matrix (ECM) alteration, and renal tubulointerstitial fibrosis (Bhxa et al., 2020). In recent years, the role of cellular senescence in DKD has gained increasing attention. Hyperglycemia accelerates the aging of glomerular mesangial cells and renal tubular epithelial cells and induces the secretion of SASP components, thus promoting inflammation and cellular senescence (Xiong and Zhou, 2019). Since the mechanisms of cellular senescence are complex, exploring its role in DKD would provide critical clues for treatment.

Metformin is a cost-effective and highly safe biguanide derivative widely used in cancer, cardiovascular disease, and kidney disease. It is one of the most commonly used drugs for the treatment of type 2 diabetes (Lv and Guo, 2020). Under the condition of high glucose, the survival rate of human umbilical vein endothelial cells decreased, and oxidative stress and chromosomal abnormalities occurred, indicating that diabetes hyperglycemia can interfere with the biochemical or biophysical properties of endothelial cells (Rezabakhsh et al., 2017). Moreover, high glucose also significantly reduced the viability of human umbilical vein endothelial cells and inhibited cell migration, while metformin significantly improved these characteristics, and promoted the angiogenic potential of endothelial cells, regulated the dynamics of endothelial cells under high glucose conditions, and improved the development of diabetic foot ulcers (Zolali et al., 2019). Furthermore, metformin alleviates the development of DKD by inhibiting the deposition of ECM and inflammation of glomerular mesangial cells by regulating the H19/miR-143-3p/TGF-β1 signaling pathway (Xu et al., 2020). It also reduces the production of oxidative stress during DKD through AMPK/SIRT1-FoxO1 pathway, enhances the autophagy response in the early stage of DKD, and reduces renal tubulointerstitial fibrosis (Ren et al., 2019; Wang et al., 2021). Metformin also exerts effects on diabetic retinopathy, aging, and cancer through mechanisms beyond nonapoptotic cell death, immunosuppression, and AMPK pathways (Hsu et al., 2021). In addition, metformin has a regulatory role in the renal aging process of DKD and delays the progress of DKD by reducing cellular senescence. Also, an active role is effectuated in high-glucose-induced renal tubular epithelial cells and *db/db* mice model through the MBNL1/miR-130a-3p/STAT3 pathway (Jiang et al., 2020). Metformin also reduces the loss of podocytes, mesangial cell apoptosis, and renal tubular epithelial cell senescence through the AMPK signal transduction pathway and exerts a renal protective role in DKD (Song et al., 2021). The anti-inflammatory and anti-fibrosis mechanisms of metformin and alleviation of cellular senescence in DKD are yet to be elucidated. Therefore, exploring the potential mechanism between metformin, DKD, and cellular senescence underlying the progression of DKD development is imperative.

Cell cycle-related protein E2F1 is a member of the E2F transcription factor family. It is involved in several biological processes, such as DNA damage response, cell migration and invasion, differentiation, metabolism, and cell cycle regulation (Roworth et al., 2015; Liang et al., 2016). E2F1 leads to hyperlipidemia and hyperglycemia in DM. Thus, inhibiting E2F1 activity prevents the hyperglycemia caused by obesity (Giralt et al., 2018). In the case of high glucose, the knockout of *E2F1* gene can save high-glucose-induced neuronal cell death (Wang et al., 2017). In addition, the regulatory effect of E2F1 on the cell cycle suggested a regulatory effect of E2F1 on cellular senescence. Moreover, P21 and P16, potent inhibitors of cell cycle-dependent kinase (CDK), blocked the phosphorylation of CDK-dependent retinoblastoma (RB) phosphorylation, leading to E2F1 inhibition and cell cycle arrest (Dick and Rubin, 2013). In addition, the overexpression of E2F1 induces senescence of normal cells (Xie et al., 2014). However, in human cancer cells, the expression of senescence markers increased after transfection with a small interference RNA (siRNA) to knock down the expression of E2F1 (Park et al., 2006). Together, these studies suggested that the regulation of E2F1 on cellular senescence is complex and unknown, and DKD is yet to be investigated.

In this study, we proved that high glucose causes renal tubular epithelial cell senescence by elevating the expression of E2F1, and metformin effectuates anti-fibrosis, reducing DNA damage response and anti-renal tubular epithelial cell aging by downregulating the expression of E2F1.

## Materials and Methods

### DM Mouse Model Groups

A total of 30 specific pathogen-free (SPF) grade male *db/db* mice, 7-week-old, weighing 40 ± 5 g and 10 non-transgenic *db/m* mice with the same background at the same age, weighing 20 ± 5 g were provided by GemPharmatech Co., Ltd. The batch number is BSK-DB T002407. The 7-week-old *db/m* mice comprised the normal control (NC) group. *db/db* mice were randomly and equally divided into DM group, AAV-shE2F1, and metformin (Met) groups. The mice were fed SPF grade feed, allowed pure water drinking *ad libitum*, and maintained at an appropriate temperature, humidity, and 12 h/day light. The AAV-shE2F1 group was injected the adeno-associated virus carrying the *E2F1* knockdown gene [Obio Technology (Shanghai) Corp. Ltd.] through the tail vein at week 16. The dose for each mouse was 2.4 × 10^11^ VG·/ml. The Met group was treated with metformin (Sino American Shanghai Squib Pharmaceutical Ltd.) by gavage at a dose of 260 mg/kg/d for 6 days/week and continuously for 8 weeks. All mice ate and drank water normally and were sacrificed at week 24. Mice urine, fasting serum, and kidney tissues were collected for the subsequent studies.

### Detection of Biochemical Indexes of Mice

Blood glucose (BG) was measured using the glucose oxidase method. Blood urea nitrogen (BUN) was measured using the urease continuous monitoring method. Serum creatinine (S-CRE) was measured via the oxidase method. Total cholesterol (T-CHO) was measured using the cholesterol oxidase method. Triglyceride (TG) was measured using the phosphoglycerol oxidase method. Urinary microalbumin was measured by enzyme-linked immunosorbent assay (ELISA).

### Histopathological Observation of Mice Kidney Tissues

The renal tissue sections were fixed with 4% neutral formaldehyde, embedded in paraffin, and stained with hematoxylin-eosin (HE), periodic acid Schiff (PAS), Masson, and Sirus red after dewaxing. The pathomorphological changes of renal tissue sections in the different groups of mice were observed under an ordinary optical microscope (OLYPUMS, Japan).

### Cell Culture, Transfection, and Administration

mRTEC cell line was obtained from Otwo Biotech Inc. (Catalogue No: HTX2460). and cultured in medium (DMEM; Gibco, United States, low glucose, containing 5.5 mmol/L glucose) containing 10% fetal bovine serum (FBS; Biological Industries, Israel) in a constant temperature incubator under 5% CO_2_ at 37°C. At 90% confluency, the cells were subcultured and used in subsequent experiments. E2F1 siRNA (Jima, Shanghai) and E2F1 overexpression plasmid (Yi le Biotech, Shanghai) were transfected into mRTECs, respectively. The negative control siRNA was transfected into the normal glucose/high glucose empty group (NG/HG + Control), the E2F1 siRNA was transfected into the normal glucose/high glucose knockdown group (NG/HG + siRNA E2F1). The cells cultured in normal glucose medium (5.5 mmol/L) and high glucose medium (30 mmol/L) containing 10% FBS were plated in a six-well plate for 72 h, followed by whole-protein extraction for subsequent experiments. Cell counting kit-8 (CCK-8; APEXBIO, United States) assay was used to detect the proliferation ability of mRTECs cultured in high-glucose after metformin (MedChemExpress, United States) intervention concentrations 10, 50, 100, and 150 μmol/L. The concentration of 50 μmol/L with maximal proliferation ability was selected for mRTEC intervention. Then, the cells were randomly divided into five groups: normal glucose/high glucose group (NG/HG), normal glucose/high glucose metformin intervention group (NG/HG + Met), high glucose metformin intervention and overexpression E2F1 plasmid transfection group (HG + Met + overexpress-E2F1) and cultured for 72 h for further experimental studies.

### Western Blot

Mice renal cortical proteins were extracted and subjected to acrylamide gel electrophoresis. The proteins were detected by incubation with rabbit anti-E2F1 (Abcam, ab112580, 1:1,000), rabbit anti-ATM (Absin, abs131163, 1:1,000), rabbit anti-p-ATM (Absin, abs140239, 1:1,000), rabbit anti-Chk2 (Bioss, bs-1391R, 1:1,000), rabbit anti-p-Chk2 (Bioss, bs-3721R, 1:1,000), rabbit anti-P21 (Proteintech, 10355-1-AP, 1:1,000), rabbit anti-Fibronectin (Abcam, ab2413, 1:1,000), rabbit anti-Collagen III (Proteintech, 22734-1-AP, 1:1,000), and β-actin antibody-horseradish peroxidase (HRP)-conjugated (PMK, PMK058S, 1:5,000) antibodies at 4°C overnight. The next day, the polyvinylidene fluoride (PVDF; Millipore, United States) membranes were incubated with secondary antibodies at room temperature for 1 h and visualized by Smart-ECL (Beyotime Biotechnology, Shanghai) chemiluminescence solution, and the immunoreactive bands were analyzed quantitatively by Image J software.

### Real-Time Polymerase Chain Reaction

Total RNA was extracted from mice renal cortex using TRIzol reagent (Ambion, Thermo, United States) and reverse transcribed into complementary DNA (cDNA). *E2F1*, *P21*, *Fibronectin*, and β-*actin* were amplified using cDNA as the template, with β-actin as an internal reference. The relative expression of *E2F1*, *P21*, and *Fibronectin* mRNA was calculated with the 2^−ΔΔCt^ method and analyzed statistically. The primer sequences (Biotech.Co., Ltd.) were as follows: *E2F1*: Forward: 5’-GAG​AAG​TCG​CGC​TAT​GAA​ACC​TC-3’, Reverse: 5’-CCC​AGT​TCA​GGT​CAA​CGA​CAC-3’ (annealing temperature 55.3°C); *P21*: Forward: 5’-TGT​CTT​GCA​CTC​TGG​TGT​CTG-3’, Reverse: 5’-ATC​TGC​GCT​TGG​AGT​GAT​AGA-3’ (annealing temperature 55.6°C); *Fibronectin*: Forward: 5’-GTC​CAT​TGA​GCT​AAC​CAA​CCT​C-3’, Reverse: 5’-GCA​GGA​GAT​TTG​TTA​GGA​CCA​C-3’ (annealing temperature 55.8°C); *β-actin*: Forward: 5’-GTC​CCT​CAC​CCT​CCC​AAA​AG-3’, Reverse: 5’-GCT​GCC​TCA​ACA​CCT​CAA​CCC-3’ (annealing temperature 58.5°C).

### Immunofluorescence of Kidney Tissue Sections

The tissue sections were dewaxed in doble distilled water (ddH_2_O) before the antigen was retrieved by heating in citric acid in a microwave oven. Then, the sections were blocked with 3% hydrogen peroxide to remove endogenous catalase, permeabilized with 0.5% Triton X-100, washed with phosphate-buffered saline (PBS), blocked with 5% bovine serum albumin (BSA) at room temperature for 1 h, and incubated with mouse anti-E2F1 antibody (1:50) and mouse anti-P21 antibody (1:50) at 4°C overnight, followed by incubation with Cy3 antibody (1:200) at 37°C in the dark for 1 h. The nucleus was stained with DAPI (Solarbio, Beijing, China), and the tissue sections were sealed with an anti-fluorescence quencher, observed, and photographed under a fluorescence microscope (OLYPUMS, Japan).

### Cellular Immunofluorescence

The cells were cultured in six-well plates and divided into groups according to the experiments. After the intervention, the cells were fixed with 4% paraformaldehyde, permeabilized with 0.5% TritonX-100, washed with PBS, and blocked with 10% BSA at 37°C for 30 min. The other steps were the same as those of tissue immunofluorescence.

### Immunohistochemistry

The procedure of tissue section dewaxing to hydrogen peroxide blocking was the same as that of tissue immunofluorescence, followed by PBS washes. Then, the sections were incubated with mouse anti-E2F1 antibody (1:50) and rabbit anti-Fibronectin antibodies (1:50) at 4°C overnight, followed by incubation with goat anti-mouse/rabbit IgG-labeled with HRP at room temperature for 20 min. Subsequently, the sections were stained with DAB [Gene Technology (Shanghai) Co., Ltd., GK500705]. The nucleus was stained with hematoxylin, dehydrated, sealed with neutral resin, observed, and photographed under an optical microscope.

### β-Galactosidase Staining

The staining procedure was carried out according to the instructions of the kit (Cell Signaling Technology). After sealing, the sections were observed and imaged captured under the optical microscope.

### Enzyme-Linked Immunosorbent Assay

The content of TNF-α and IL-6 in mouse tissue supernatant was detected using the Elabscience kit.

### Network Pharmacology Analysis

The targets of metformin were obtained through relevant databases. The relevant targets of DKD were screened from the GeneCards database. The Venn diagram program obtained the putative targets of drugs for disease treatment. Metascape platform was used to annotate the intersecting proteins through gene ontology (GO). Protein-protein interaction (PPI) pathways were used to analyze the intersection proteins using the STRING database and Cytoscape software.

### Statistical Analysis

The Western blot images were analyzed by Image J software. The experimental data were analyzed by SPSS 25.0 software and expressed as mean ± standard deviation (SD); one-way ANOVA was used for comparison among multiple groups, LSD test was used for comparison between the two groups, *p* < 0.05 indicated a statistically significant difference.

## Results

### Results of Network Pharmacology

**FIGURE 1:**
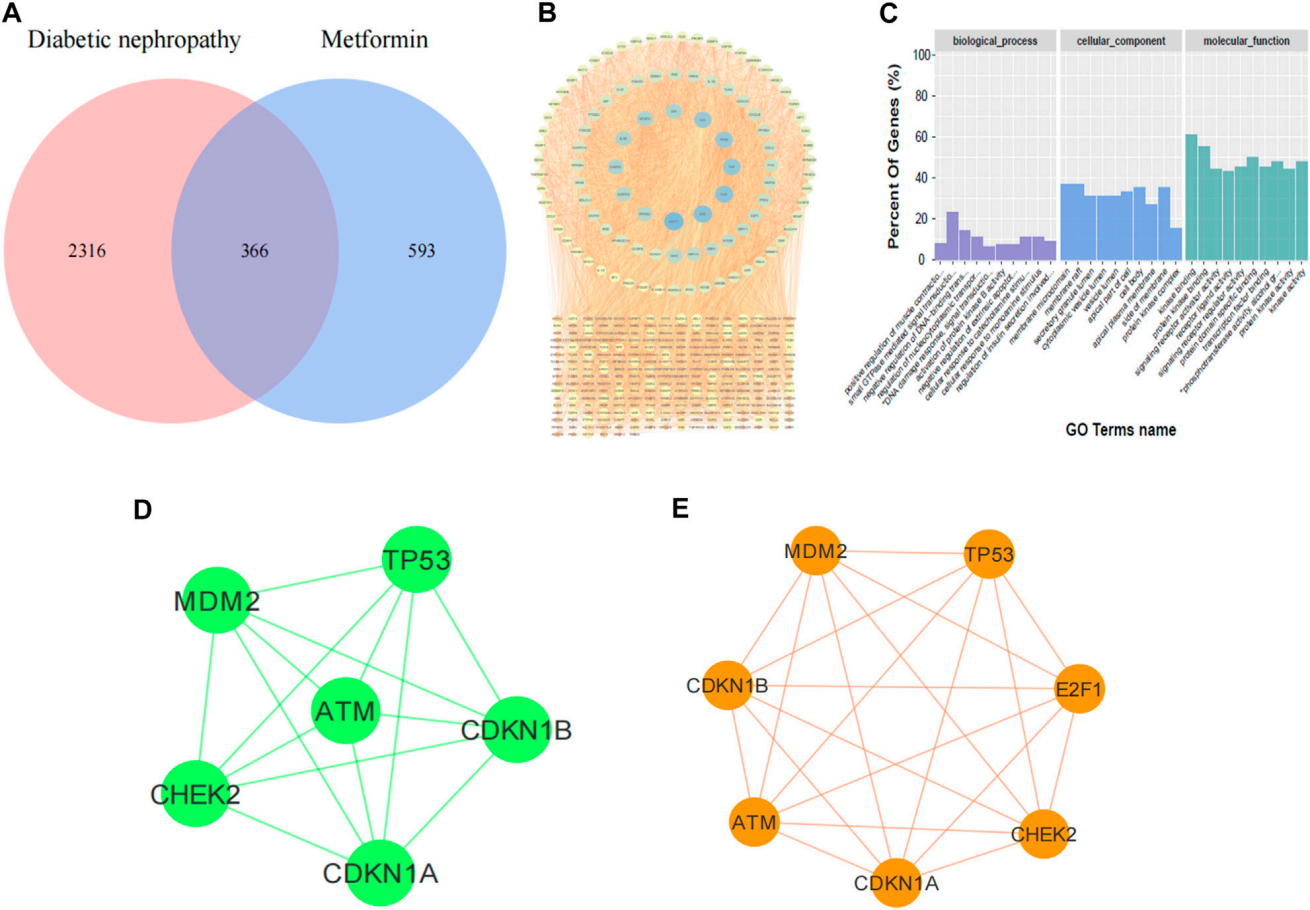
Results of network pharmacology prediction. **(A)** Venn diagram of DKD**-**metformin target intersection. **(B)** PPI diagram of intersection protein. **(C)** Diagram of GO enrichment analysis. **(D)** PPI diagram of metformin treatment against DKD by regulating DNA damage. **(E)** PPI diagram of E2F1 involved in DNA damage in DKD treated with metformin.

In order to identify the targets of metformin in the treatment of DKD, we retrieved 366 coincident targets of metformin and DKD from the database (Figure 1A). These coincident targets are putative targets of metformin in the treatment of DKD. The PPI network diagram was constructed for all proteins at the intersection (Figure 1B). The results of the GO enrichment analysis showed that biological processes, such as aging and DNA damage, were involved in identifying the potential targets of metformin in the treatment of DKD (Figure 1C). Therefore, the PPI network diagram of this process was constructed separately (Figure 1D). The core targets include ATM, Chk2, CDKN1A (P21), CDKN1B, MDM2, and TP53. Finally, the altered expression of P21, ATM, and Chk2 was observed in the subsequent experiments. Then, bioinformatics was utilized to identify the proteins that directly interact with E2F1. These were combined with the network pharmacology prediction targets and E2F1 interaction protein to construct the PPI network diagram (Figure 1E). The results showed that E2F1 was directly involved in the process of DNA damage in the treatment of DKD with metformin.

### Changes in the Biochemical Indexes of Mice in Each Group

Compared to the NC group, BG, TG, T-CHO, S-CRE, BUN, and urine microalbumin levels were significantly increased in the DM group. Compared to the DM group, the above indexes in the Met group showed an opposite trend, the levels of BG, BUN, and urine microalbumin were significantly decreased in the AAV-shE2F1 group, the levels of TG, cholesterol-CHO and S-CRE were no significant changes. (*p* < 0.05; Table 1).

**TABLE 1:** Changes in BG, TG, T-CHO, S-CRE, BUN, and urine microalbumin of mice in each group (n = 6,^*^ *p* < 0.05 vs. NC.^#^ *p* < 0.05 vs. DM).

Group	NC	DM	Met	AAV-shE2F1
BG (mmol/L)	5.57 ± 1.35	23.25 ± 1.85^*^	13.39 ± 3.94^#^	16.11 ± 1.72^#^
TG (mmol/L)	1.09 ± 0.43	1.91 ± 0.44^*^	1.20 ± 0.20^#^	1.78 ± 0.32
T-CHO (mmol/L)	1.73 ± 0.64	5.18 ± 0.89^*^	1.84 ± 0.71^#^	4.78 ± 0.48
S-CRE (μmol/L)	21.28 ± 3.02	51.49 ± 10.74^*^	23.06 ± 8.66^#^	43.84 ± 7.48
BUN (mmol/L)	2.92 ± 0.82	8.68 ± 1.48^*^	4.43 ± 0.81^#^	4.66 ± 0.36^#^
MAU (ng/ml)	8.52 ± 1.79	131.89 ± 17.08^*^	21.02 ± 2.46^#^	27.27 ± 5.77^#^

### Pathomorphological Changes in the Renal Tissue in Each Group

**FIGURE 2:**
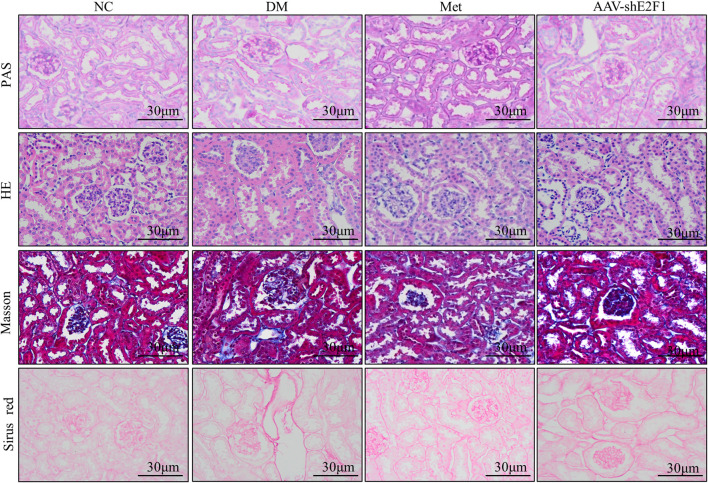
Renal histopathological changes of mice in each group (PAS, HE, Masson, and Sirus red staining).

The results of PAS staining showed that the basement membrane of glomerulus and renal tubules was significantly thickened in the DM group compared to the NC group, and the mesangial area was accompanied by cell proliferation. The results of HE staining showed that the glomerulus and renal tubules of the NC group mice had complete structure and regular morphology, while those of the DM group had glomerulosclerosis, renal tubular atrophy, and epithelial cell abscission. Masson and Sirus red staining showed that collagen fibers were deposited in the renal interstitium of the DM group, while the above pathological changes were alleviated in the AAV-shE2F1 and Met groups (Figure 2).

### Metformin Improves the Process of Renal Fibrosis and the Degree of DNA Damage and Cellular Senescence in the High-Glucose State

**FIGURE 3:**
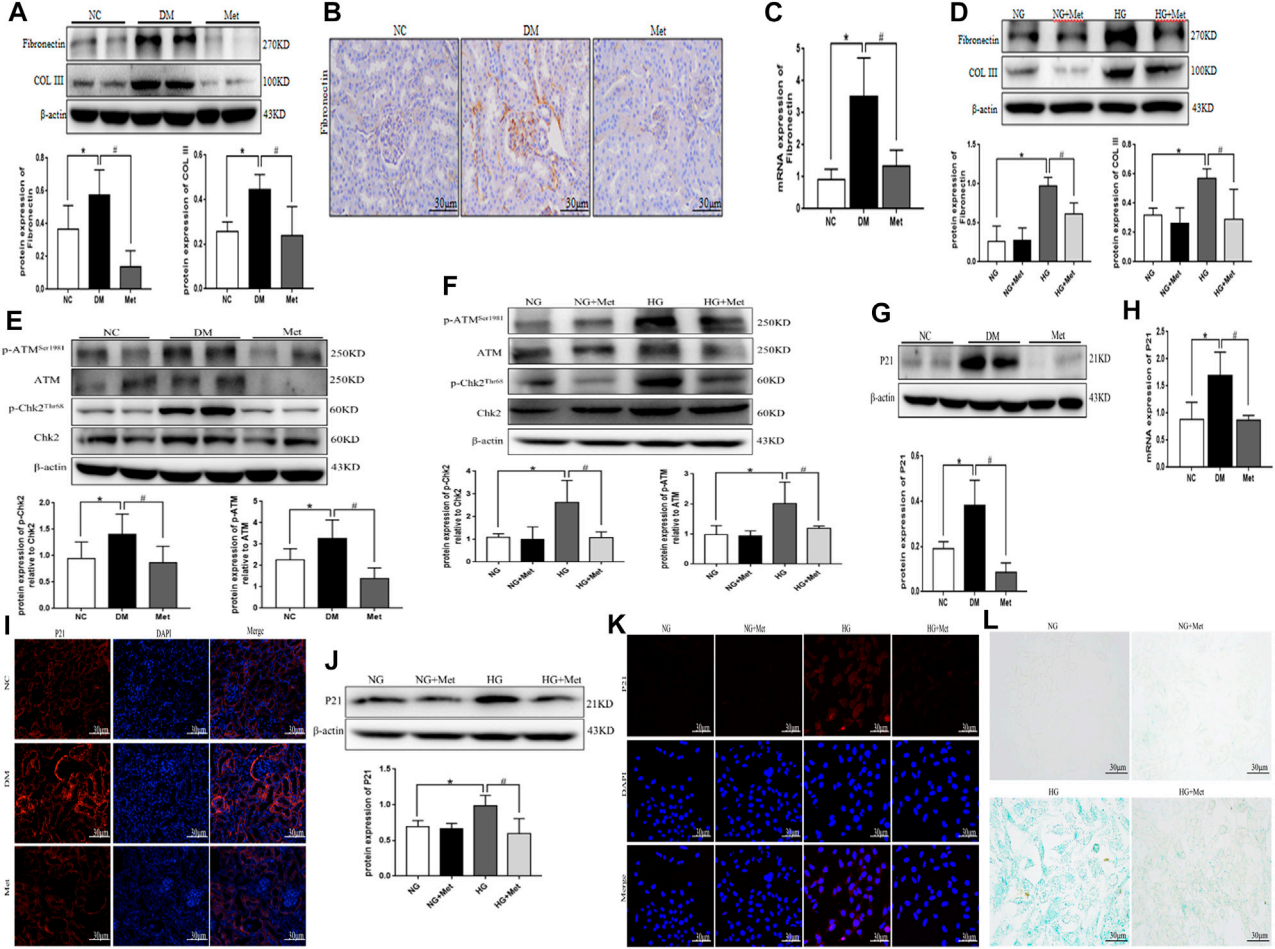
Metformin improves the process of renal fibrosis and the degree of DNA damage and cellular senescence in the high-glucose state. **(A)** Western blot analysis of Fibronectin and COL III in renal cortex of mice in each group (n = 6, ^*^ *p* < 0.05 vs. NC, ^#^ *p* < 0.05 vs. DM). **(B)** Immunohistochemical staining of Fibronectin in renal cortex of mice in each group. **(C)** Real-time PCR analysis of Fibronectin in renal cortex of mice in each group (n = 6, ^*^ *p* < 0.05 vs. NC, ^#^ *p* < 0.05 vs. DM). **(D)** Western blot analysis of Fibronectin and COL III in mRTECs (n = 3, ^*^ *p* < 0.05 vs. NG, ^#^ *p* < 0.05 vs. HG). **(E)** Western blot analysis of ATM, p-ATM^Ser1981^, Chk2, and p-Chk2^Thr68^ in renal cortex of mice in each group (n = 6, ^*^ *p* < 0.05 vs. NC, ^#^ *p* < 0.05 vs. DM). **(F)** Western blot analysis of ATM, p-ATM^Ser1981^, Chk2, and p-Chk2^Thr68^ in mRTECs (n = 3, ^*^ *p* < 0.05 vs. NG, ^#^ *p* < 0.05 vs. HG). **(G)** Western blot analysis of P21 in the renal cortex of mice in each group (n = 6, ^*^ *p* < 0.05 vs. NC, ^#^ *p* < 0.05 vs. DM). **(H)** Real-time PCR analysis of P21 in the renal cortex of mice in each group (n = 6, ^*^ *p* < 0.05 vs. NC, ^#^ *p* < 0.05 vs. DM). **(I)** Tissue immunofluorescence of P21 in renal cortex of mice in each group. **(J)** Western blot analysis of P21 in mRTECs (n = 3, ^*^ *p* < 0.05 vs. NG, ^#^ *p* < 0.05 vs. HG). **(K)** Cellular immunofluorescence of P21 in mRTECs. **(L)** β-Galactosidase staining in mRTECs.

Western blot results showed that the protein expression levels of Fibronectin and COL III were significantly higher in the DM group than in the NC group (Figure 3A). Immunohistochemical staining and real-time PCR results showed that the protein and mRNA levels of Fibronectin were significantly higher in the DM group than in the NC group (Figures 3B,C). After metformin intervention, the expression of the above indexes decreased markedly. Compared to the NG group, the levels of Fibronectin and COL III proteins were significantly higher in the HG group, compared to the HG group, the levels of Fibronectin and COL III proteins were significantly lower in the HG + Met group (Figure 3D). The results of Western blot showed that the phosphorylation levels of DNA damage-related indexes ATM and Chk2 were significantly increased *in vitro* and *in vivo* in the high-glucose environment. The Metformin intervention significantly decreased the expression of the above indexes (Figures 3E,F). Western blot, real-time PCR, and tissue immunofluorescence showed that the level of P21 was higher in the DM group than in the NC group (Figures 3G–I). According to ELISA, the content of IL-6 and TNF-α was increased markedly in the DM group (Table 2), after metformin intervention, the expression of the above indexes decreased markedly. Western blot and cellular immunofluorescence results showed that the expression of P21 was significantly higher in the HG group than the NG group and decreased significantly in the HG + Met group (both *p* < 0.05; Figures 3J,K). Interestingly, the β-galactosidase-positive staining was significantly more in the HG group than the NG group but significantly reduced in the HG + Met group (Figure 3L). These results suggested that metformin improves the process of renal fibrosis in the high-glucose state, reduces the degree of DNA damage, and delays cellular senescence.

**TABLE 2:** Content of IL-6 and TNF-α in the renal tissue supernatant of mice in each group (n = 6,^*^ *p* < 0.05 vs. NC.^#^ *p* < 0.05 vs. DM).

Group	IL-6	TNF-α
NC	317.58 ± 178.71	228.76 ± 158.99
DM	505.74 ± 151.15^*^	482.26 ± 206.97^*^
Met	376.32 ± 71.57^#^	272.56 ± 126.05^#^

### High Expression of E2F1 in Renal Tubular Epithelial Cells in the High-Glucose State

**FIGURE 4:**
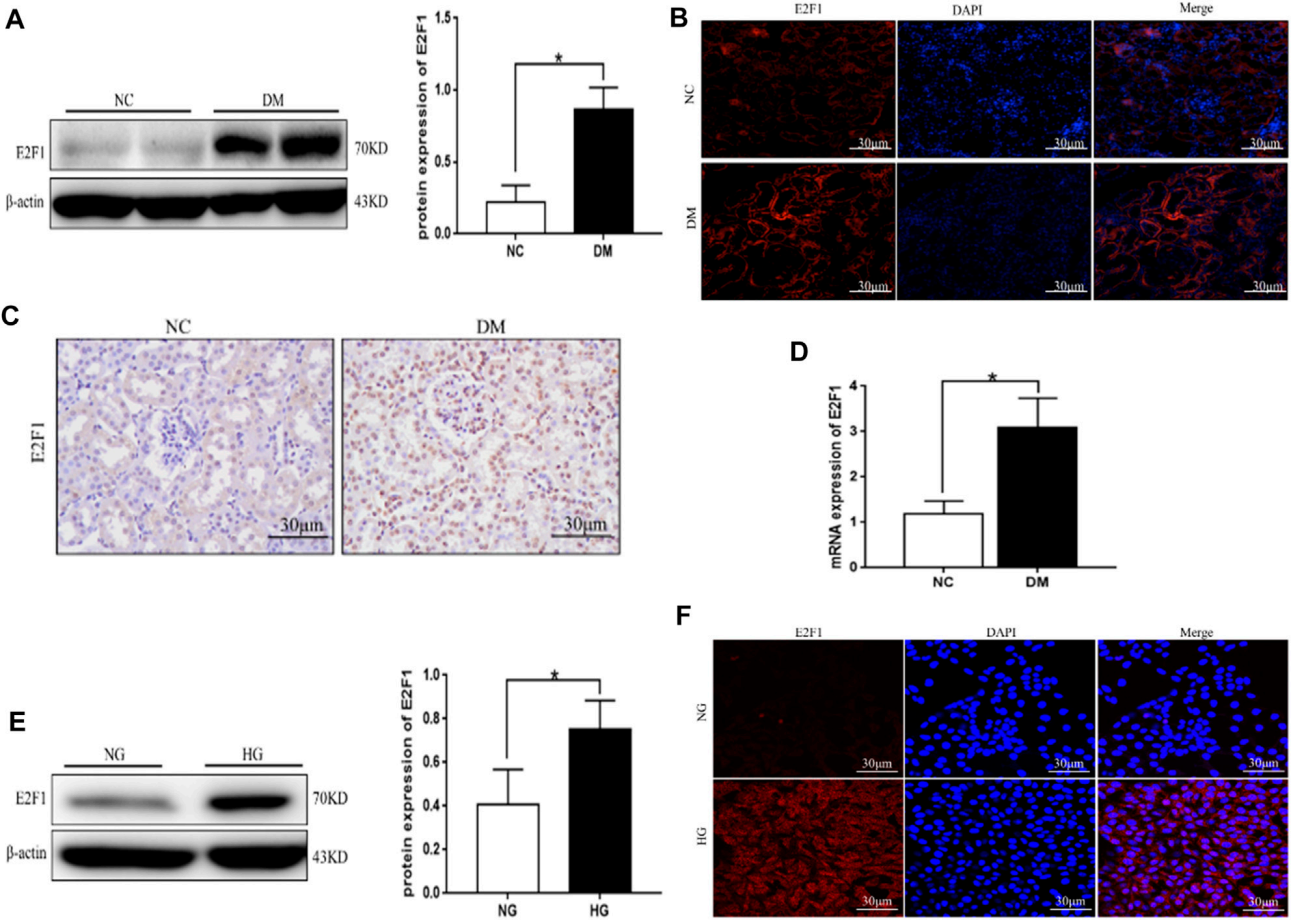
High expression of E2F1 was observed in renal tubular epithelial cells in the high-glucose state. **(A)** Western blot analysis of E2F1 in the renal cortex of mice in each group (n = 6, ^*^ *p* < 0.05 vs. NC). **(B)** Tissue immunofluorescence of E2F1 in the renal cortex of mice in each group. **(C)** Immunohistochemical staining of E2F1 in the renal cortex of mice in each group. **(D)** Real-time PCR analysis of E2F1 in the renal cortex of mice in each group (n = 6, ^*^ *p* < 0.05 vs. NC). **(E)** Western blot analysis of E2F1 in mRTECs (n = 3, ^*^ *p* < 0.05 vs. NG). **(F)** Cellular immunofluorescence of E2F1 in mRTECs.

Western blot, tissue immunofluorescence, immunohistochemical staining, and real-time PCR showed significantly higher expression of E2F1 in the DM group than the NC group (Figures 4A–D). Western blot and cellular immunofluorescence results showed that the expression of E2F1 was significantly higher in the HG group than in the NG group (*p* < 0.05; Figures 4E,F).

### High Expression of E2F1 Promotes the Fibrosis of Renal Tubular Epithelial Cells in the High-Glucose State

**FIGURE 5:**
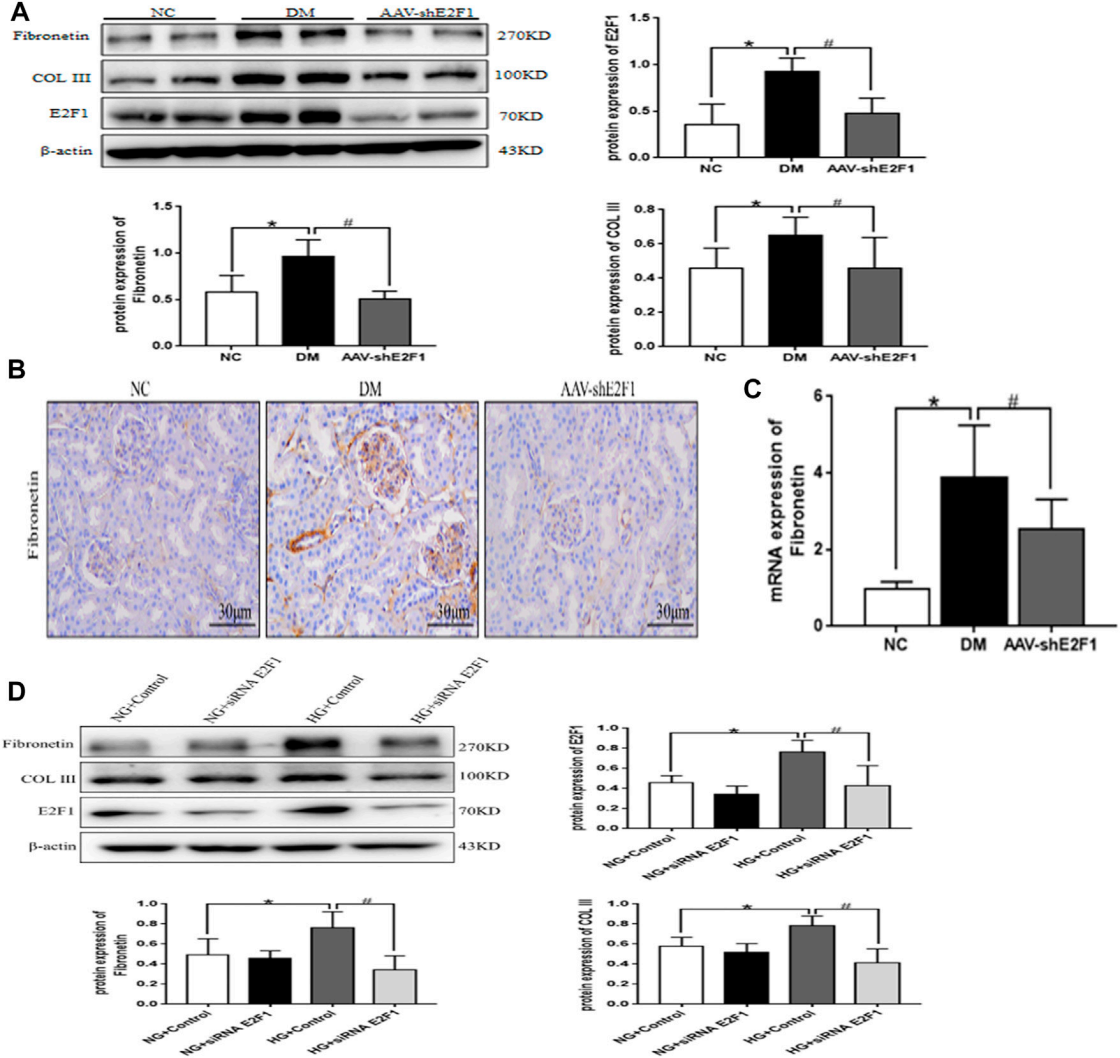
High-expression of E2F1 promotes the fibrosis of renal tubular epithelial cells in the high-glucose state. **(A)** Western blot analysis of E2F1, Fibronectin, and COL III in the renal cortex of mice in each group (n = 6, ^*^ *p* < 0.05 vs. NC, ^#^ *p* < 0.05 vs. DM). **(B)** Immunohistochemical staining of Fibronectin in the renal cortex of mice in each group. **(C)** Real-time PCR analysis of Fibronectin in the renal cortex of mice in each group (n = 6, ^*^ *p* < 0.05 vs. NC, ^#^ *p* < 0.05 vs. DM). **(D)** Western blot analysis of E2F1, Fibronectin, and COL III in mRTECs (n = 3, ^*^ *p* < 0.05 vs. NG + Control, ^#^ *p* < 0.05 vs. HG + Control).

Western blot showed that the levels of Fibronectin and COL III proteins were significantly higher in the DM group than in the NC group (Figure 5A). Immunohistochemical staining and real-time PCR revealed that the protein and mRNA levels of Fibronectin were significantly higher in the DM group than those in the NC group (Figures 5B,C). However, the expression of the above indexes was significantly decreased in the AAV-shE2F1 group. Compared to the NG + Control and NG + siRNA E2F1 groups. The protein levels of Fibronectin and COL III were significantly higher in the HG group and significantly lower in the HG + siRNA E2F1 group than those in the HG group (*p* < 0.05; Figure 5D). Thus, the high expression of E2F1 can promote the fibrosis of renal tubular epithelial cells in the high-glucose state and downregulating the expression can improve the process of fibrosis.

### High Expression of E2F1 Promotes the Degree of DNA Damage in Renal Tubular Epithelial Cells in the High-Glucose State

**FIGURE 6:**
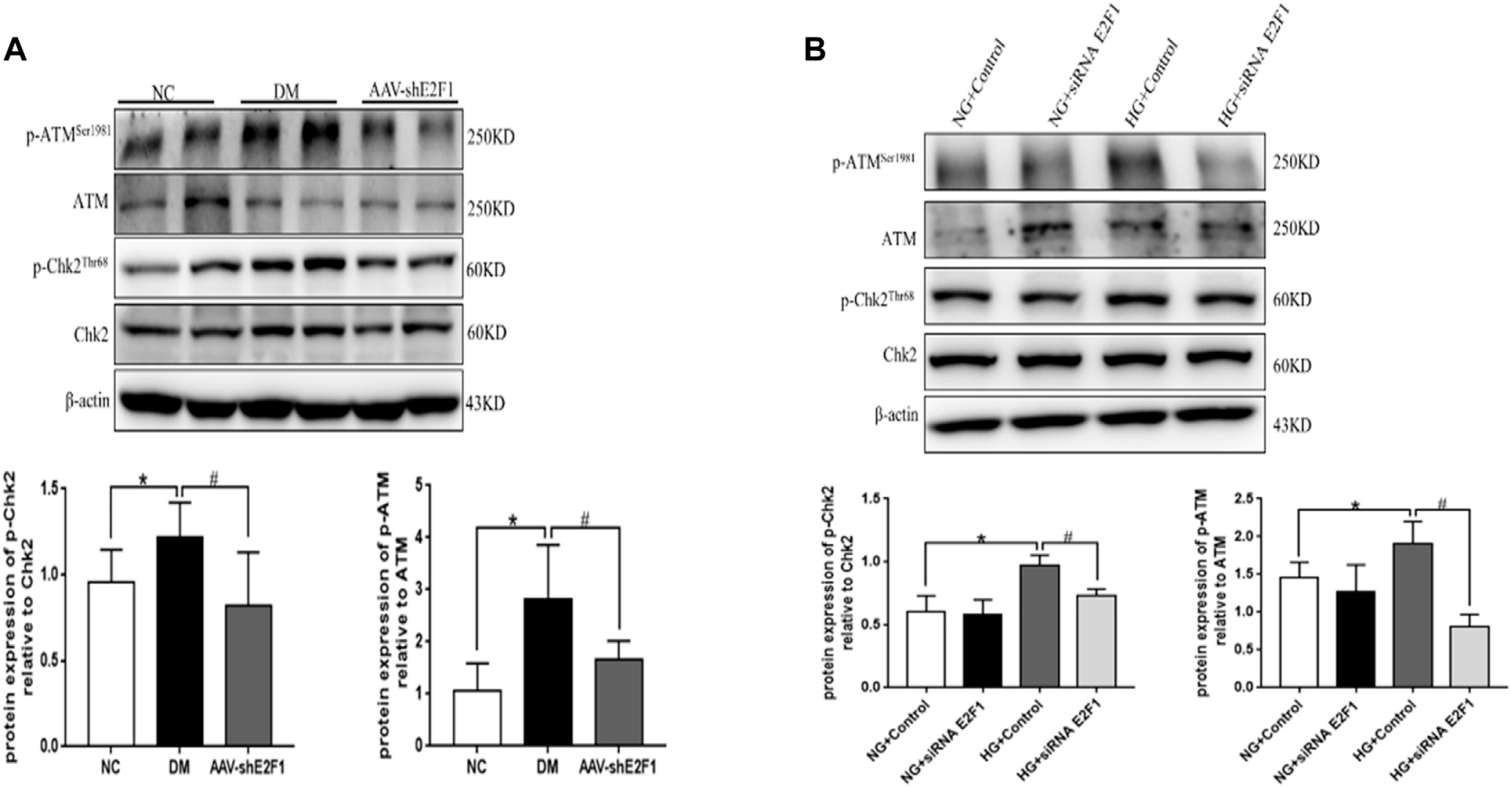
High expression of E2F1 promotes DN damage in renal tubular epithelial cells in the high-glucose state. **(A)** Western blot analysis of ATM, p-ATM^Ser1981^, Chk2, and p-Chk2^Thr68^ in the renal cortex of mice in each group (n = 6, ^*^ *p* < 0.05 vs. NC, ^#^ *p* < 0.05 vs. DM). **(B)** Western blot analysis of ATM, p-ATM^Ser1981^, Chk2, and p-Chk2^Thr68^ in mRTECs (n = 3, ^*^ *p* < 0.05 vs. NG + Control, ^#^ *p* < 0.05 vs. HG + Control).

The phosphorylation levels of DNA damage-related indexes, ATM and Chk2, were significantly increased *in vitro* and *in vivo* under the high-glucose environment but reduced after the knockdown of E2F1 (*p* < 0.05; Figures 6A,B). This phenomenon suggested that high expression of E2F1 aggravates the DNA damage of renal tubular epithelial cells in the high-glucose state.

### High Expression of E2F1 Promotes Cellular Senescence of Renal Tubular Epithelial Cells in the High-Glucose State

**FIGURE 7:**
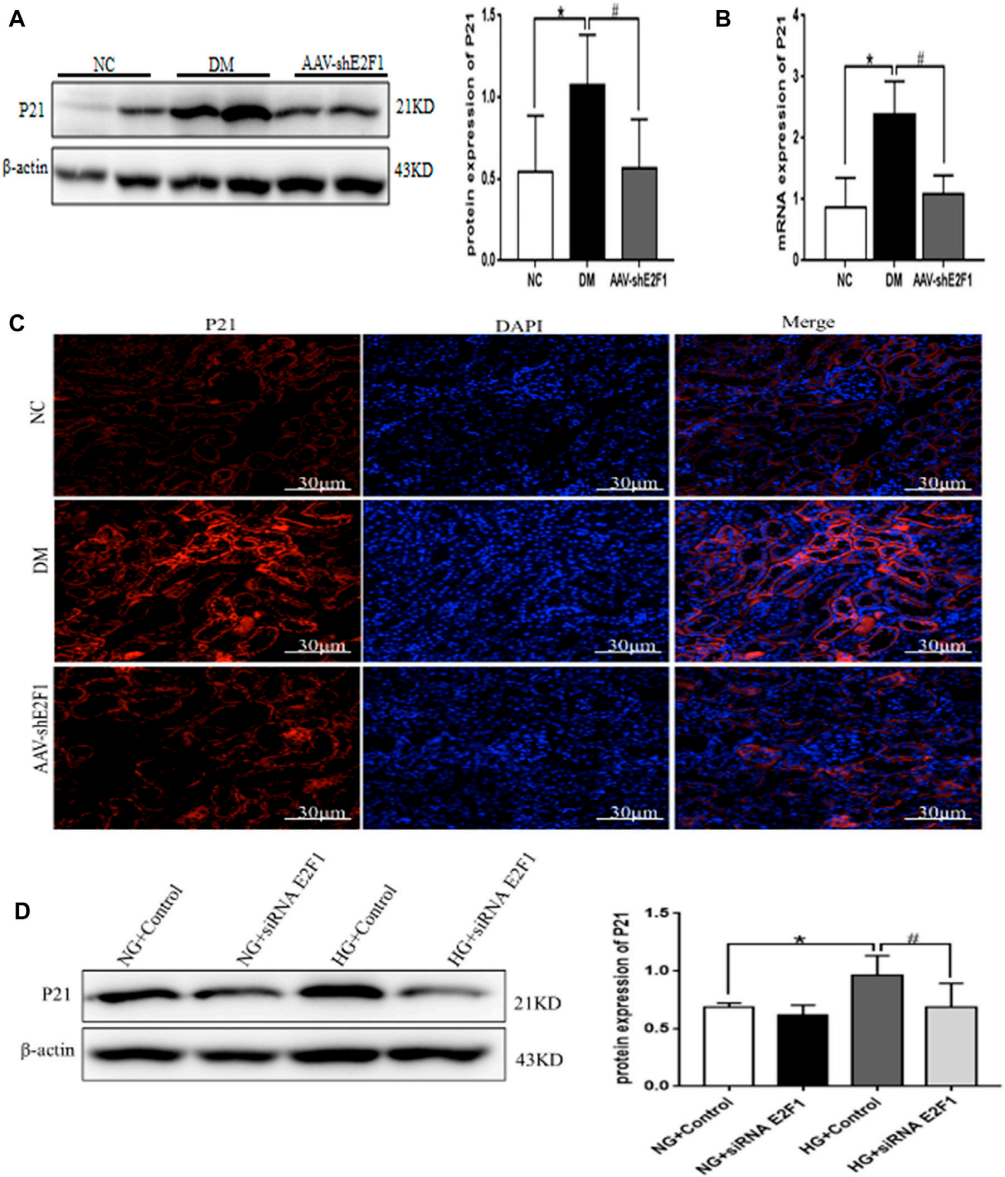
High expression of E2F1 promotes cellular senescence of renal tubular epithelial cells in the high-glucose state. **(A)** Western blot analysis of P21 in the renal cortex of mice in each group (n = 6, ^*^ *p* < 0.05 vs. NC, ^#^ *p* < 0.05 vs. DM). **(B)** Real-time PCR analysis of P21 in the renal cortex of mice in each group (n = 6, ^*^ *p* < 0.05 vs. NC, ^#^ *p* < 0.05 vs. DM). **(C)** Tissue immunofluorescence of P21 in the renal cortex of mice in each group. **(D)** Western blot analysis of P21 in mRTECs (n = 3, ^*^ *p* < 0.05 vs. NG + Control, ^#^ *p* < 0.05 vs. HG + Control).

Western blot, real-time PCR, and tissue immunofluorescence showed that the expression of P21 was significantly higher in the DM group than the NC group and lower in the AAV-shE2F1 group (Figures 7A–C). The results of ELISA revealed that the content of IL-6 and TNF-α was higher in the DM group and significantly lower in the AAV-shE2F1 group than in the NC group (Table 3). Compared to the NG + Control and NG + siRNA E2F1 groups, the level of P21 protein was significantly higher in the HG group and significantly lower in the HG + siRNA E2F1 group (*p* < 0.05; Figure 7D). This phenomenon suggested that the high expression of E2F1 promotes the senescence of renal tubular epithelial cells in the high-glucose state.

**TABLE 3:** Content of IL-6 and TNF-α in renal tissue supernatant of mice in each group (n = 6,^*^ *p* < 0.05 vs. NC.^#^ *p* < 0.05 vs. DM).

Group	IL-6	TNF-α
NC	303.36 ± 125.87	236.52 ± 150.77
DM	510.29 ± 146.11^*^	485.83 ± 203.90*
AAV-shE2F1	326.41 ± 66.86^#^	273.13 ± 81.37^#^

### Metformin Improves Renal Injury in the High-Glucose State Through E2F1

**FIGURE 8:**
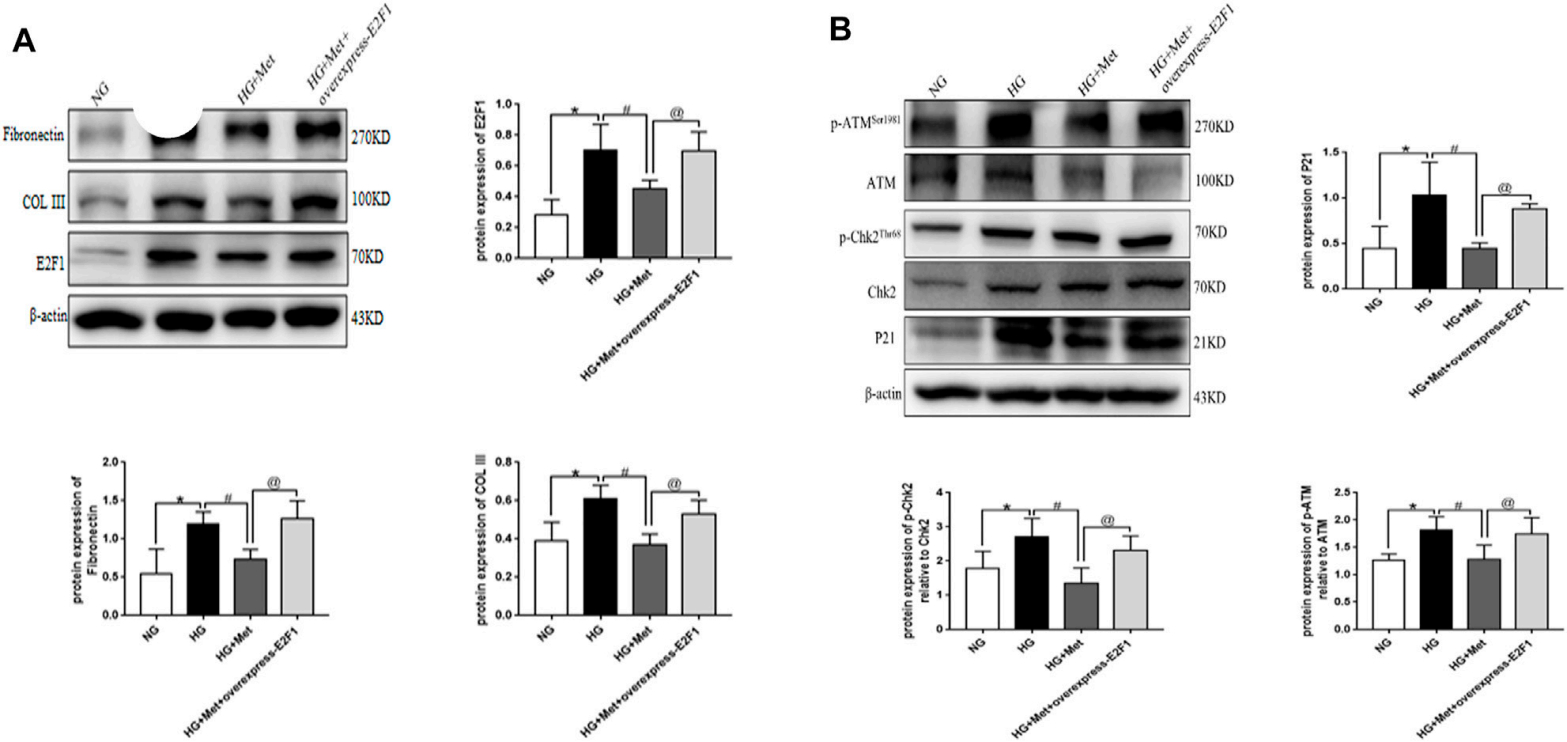
Metformin improves renal injury in the high-glucose state through E2F1. **(A)** Western blot analysis of E2F1, Fibronectin, and COL III in mRTECs (n = 3, ^*^ *p* < 0.05 vs. NG, ^#^ *p* < 0.05 vs. HG, ^@^ *p* < 0.05 vs. HG + Met). **(B)** Western blot analysis of ATM, p-ATM^Ser1981^, Chk2, and p-Chk2^Thr68^ in mRTECs (n = 3, ^*^ *p* < 0.05 vs. NG, ^#^ *p* < 0.05 vs. HG, ^@^ *p* < 0.05 vs. HG + Met).

Compared to the NG group, the expression of E2F1, Fibronectin, and COL III, the phosphorylation levels of ATM and Chk2, and the expression of P21 was significantly increased in the HG group. After the intervention with metformin, the expression of the above indexes decreased, and the consequent overexpression of E2F1 weakened the effect of metformin on the above indexes in mRTECs (Figures 8A,B, *p* < 0.05). Thus, the anti-fibrosis, anti-senescence, and DNA damage repair effects of metformin in the high-glucose state were achieved by downregulating the expression of E2F1.

## Discussion

P16 expression and SA-β-Gal activity increased in renal tubular epithelial cells, podocytes, mesangial cells, and endothelial cells in patients with type 2 DKD (Verzola et al., 2008). Also, cellular senescence markers were produced in mesangial cells cultured *in vitro* with high glucose (Zhang et al., 2006) and streptozotocin-induced mice model of type 1 diabetes (Kitada et al., 2014). These results suggested that hyperglycemia is a key driver of cellular senescence and may contribute to DKD progression. Accumulating evidence showed that cellular senescence might be involved in the pathophysiological process of DKD. A large number of studies reported the putative mechanism of cellular senescence in DKD (Chen et al., 2018; Yi-Chun et al., 2018). However, because the pathogenesis of DKD and cellular senescence is complex, the correlation and regulatory mechanism between DKD and cellular senescence need to be explored further. In the current study, we used *db/db* mice model in the *in vivo* experiments, and the model is a widely used animal model of type 2 DKD. It is a leptin receptor mutation that causes abnormal splicing and adipocyte-derived hormone leptin receptor defects, and the leptin receptor deficiency affects the hypothalamic reaction, which makes the mice appear hyperappetite, obese, hyperlipidemia, and insulin resistant, and the other symptoms are similar to those of patients with type 2 diabetes (Tesch and Lim, 2011). The results of this experiment showed that BG, TG, T-CHO, S-CRE, BUN, and urine microalbumin were significantly increased in *db/db* mice compared to *db/m* mice. HE and PAS staining revealed that the glomerular basement membrane of *db/db* mice was thickened, the mesangial area was accompanied by apparent mesangial cell proliferation, the morphology of renal tubules was irregular, and the epithelial cells of renal tubules were detached. Masson and Sirus red staining showed fibrosis in renal tubulointerstitium. All the above results are consistent with the characteristics of DKD, indicating that type 2 diabetes progressed to DKD in the *db/db* mice model.

Metformin is the most widely used oral hypoglycemic drug, which reduces DKD by inhibiting renal inflammation, oxidative stress, and fibrosis (Kawanami et al., 2020). It also reduces the senescence of renal tubular epithelial cells induced by high glucose, inhibiting high-glucose-induced renal lesions, and plays a renal protective role in DKD (Jiang et al., 2020). Herein, we searched the database to retrieve the putative targets of metformin in the treatment of DKD, involved in aging and DNA damage; the core targets include ATM, CHK2, P21, CDKN1B, MDM2, and TP53. We focused on the altered P21, ATM, and CHK2 expression in renal tubular epithelial cells in the high-glucose state. After 8 weeks of *in vivo* intervention in *db/db* mice, the results showed that metformin reduces BG, TG, T-CHO, S-CRE, BUN, and urinary microalbumin. It could also improve the pathomorphological changes of *db/db* mice. The expression of Fibronectin, COL III, and P21 proteins was decreased in the renal cortex of *db/db* mice, and inhibited the phosphorylation levels of ATM and Chk2, while the content of IL-6 and TNF-α in renal supernatant of *db/db* mice was reduced. This finding suggested that metformin improves the degree of renal tissue fibrosis, inflammation, DNA damage, and cellular senescence in DKD.

E2F1 is involved in cell cycle regulation, apoptosis signal transduction, cell growth, metastasis, autophagy, and other processes (Yuan et al., 2018). The study demonstrated the specific biological function of E2F1 in the progression and related complications of DM. It also promotes hyperlipidemia and hyperglycemia in DM by directly controlling hepatic gluconeogenesis (Giralt et al., 2018). In recent years, the role of E2F1 in cellular senescence has been under intensive focus (Gao et al., 2019). So what is the role of E2F1 in the cellular senescence in DKD? Thus, we used bioinformatics to identify the proteins acting directly with E2F1. The results suggested that E2F1 is directly involved in DNA damage and cellular senescence with respect to the treatment of DKD with metformin. Hence, we injected the adeno-associated virus carrying the *E2F1* knockdown gene into *db/db* mice through the tail vein; animals were sacrificed after 8 weeks. Consequently, the levels of BG, BUN, and urinary microalbumin of adeno-associated virus mice injected with knockdown *E2F1* gene were significantly lower than those of *db/db* mice, the levels of TG, cholesterol-CHO and S-CRE were no significant changes. Moreover, the pathological morphology was improved, and the indexes related to fibrosis, DNA damage, and cellular senescence were reduced, while the levels of IL-6 and TNF-α were significantly reduced in the supernatant of renal tissue. Interestingly, the decrease in E2F1 alleviates the degree of renal tissue fibrosis, the level of inflammation, and the senescence of renal tubular epithelial cells in DKD. In addition, metformin reduces the expression level of E2F1 in the renal tissue of *db/db* mice. To further confirm that E2F1 is directly involved in metformin-alleviated fibrosis, DNA damage, and cellular senescence in DKD, we stimulated mRTECs with high glucose *in vitro* and found that the indexes related to E2F1, fibrosis, DNA damage, and cellular senescence increased significantly, but decreased after transfection with E2F1 siRNA. This phenomenon suggested that the increase in E2F1 affects the occurrence of fibrosis and cellular senescence in DKD, which is consistent with the results *in vivo*. Moreover, the stimulation of mRTECs with high glucose and metformin demonstrated that metformin reduces the expression of E2F1 in the high-glucose state and reverses the increased collagen and fibronectin levels, DNA damage, and senescence-associated markers. Therefore, it could be deduced that metformin exerts an anti-fibrosis role and delays cellular senescence by reducing the expression of E2F1 in the high-glucose state. In order to test this hypothesis, we overexpressed E2F1 after metformin treatment in the high-glucose state. Compared to the metformin treatment alone group, the anti-fibrosis and delayed cell senescence effects of metformin after overexpression of E2F1 were significantly weakened. Together with the *in vivo* results, it was confirmed that metformin is a renal anti-fibrosis and cell aging agent in DKD via decreased expression of E2F1 in the high-glucose state.

In conclusion, the current results suggested that metformin has a protective effect on high glucose-induced renal tubular epithelial cell injury. The mechanism is to reverse the degree of fibrosis of DKD renal tissue, reduce DNA damage, and slow down the aging process of renal tubular epithelial cells by reducing the expression of E2F1. Thus, this study provided a new experimental basis for the mechanism of metformin in renal protection in DKD. It also provides new drug targets for delaying the process and treatment of renal fibrosis and cellular senescence in DKD.

## Data Availability

The original contributions presented in the study are included in the article/Supplementary Materials, further inquiries can be directed to the corresponding authors.
